# The Significance and Power of Community: Black Dementia Caregivers Navigating Care Systems

**DOI:** 10.1093/geroni/igaf122.4271

**Published:** 2025-12-31

**Authors:** Janelle Gore, Mayra Sainz, Larry Strickland, Stephanie Bennett, Carolyn Clevenger, Fayron Epps

**Affiliations:** The University of Alabama, Tuscaloosa, Alabama, United States; Emory University, Atlanta, Georgia, United States; UT Health San Antonio, San Antonio, Texas, United States; Emory University, Atlanta, Georgia, United States; Emory University, Atlanta, Georgia, United States; UT Health San Antonio, San Antonio, Texas, United States

## Abstract

As the prevalence of Alzheimer’s Disease and related dementia continues to disproportionately affect the Black community, the need to support caregivers is also expected to increase. A challenge many Black dementia caregivers face is navigating care systems due to the complexities of healthcare in the US. These difficulties can increase levels of isolation, in turn affecting the quality of care provided to their family member living with dementia. Thus, a psychoeducation course, Caregiving While Black LIVE, was developed to foster a community of Black health professionals and caregivers to equip and empower Black dementia caregivers with the sense of mastery needed to navigate care systems while acknowledging the social and cultural realities. Caregivers participated in the 6-week course, led by facilitators, meeting weekly with a group of 6 to 15 Black dementia caregivers either via Zoom or in person. Individual qualitative interviews were conducted with 17 of the 40 study participants to explore their experiences in the course. Most caregivers were women, with an average age of 57. Analysis revealed that participating in a culturally tailored psychoeducational program influences Black dementia caregivers’ experiences navigating care systems by 1. Enhancing understanding, trust, and emotional comfort, 2. Empowering ability to make informed decisions, 3. Redefining the caregiving role, and 4. Sharing knowledge within the community. Culturally tailored programming remains a critical need for Black dementia caregivers. Researchers should work to increase the development of spaces that promote communal learning to boost confidence and improve health outcomes for families affected by dementia.

